# 5-HT Drives Mortality in Sepsis Induced by Cecal Ligation and Puncture in Mice

**DOI:** 10.1155/2017/6374283

**Published:** 2017-06-13

**Authors:** Jingyao Zhang, Jianbin Bi, Sushun Liu, Qing Pang, Ruiyao Zhang, Shun Wang, Chang Liu

**Affiliations:** ^1^Department of Hepatobiliary Surgery, The First Affiliated Hospital of Xi'an Jiaotong University, Xi'an, China; ^2^Department of Surgical Intensive Care Unit, The First Affiliated Hospital of Xi'an Jiaotong University, Xi'an, China; ^3^Department of Cardiovascular Medicine, The First Affiliated Hospital of Xi'an Jiaotong University, Xi'an, China

## Abstract

Sepsis is defined as a life-threatening organ dysfunction caused by a dysregulated host response to infection with a high mortality. 5-Hydroxytryptamine (5-HT) is an important regulatory factor in inflammation. The aim of this study is to investigate the role of 5-HT on cecal ligation and puncture- (CLP-) induced sepsis in the mouse model. CLP was performed on C57B/6 wild-type (WT) mice and *tryptophan hydroxylase 1* (*TPH1*) knockout (KO) mice. The results showed that the 5-HT-sufficient group mice had a significantly lower survival rate than the 5-HT-deficient group in CLP-induced sepsis and septic shock. The KO-CLP sepsis group received a lower clinical score than the WT-CLP sepsis group. Meanwhile, the body temperature of mice in the KO-CLP sepsis group was higher than that in the WT-CLP sepsis group and was much closer to the normal body temperature 24 hours after CLP. The tissue histopathology analysis revealed that 5-HT markedly exacerbated histological damages in the peritoneum, lung, liver, kidney, intestinal tissue, and heart in sepsis. Moreover, significant lower levels of TNF-*α*, IL-6, bacterial loads, MPO, and ROS were discovered in the KO-CLP sepsis group in contrast to the WT-CLP sepsis group. In conclusion, 5-HT drives mortality and exacerbates organ dysfunction by promoting serum cytokines and bacterial loads as well as facilitating oxidative stress in the process of sepsis.

## 1. Introduction

Sepsis is defined as a life-threatening organ dysfunction caused by a dysregulated host response to infection. It is characterized by high mortality between 28% and 50% and limited therapeutic options [1–3]. In many diseases, the severe infections result in sepsis which generates serious complications including septic shock, multiple organ dysfunction syndrome (MODS), multiple organ failure, and even death [4, 5]. In the process of sepsis, the infection or bacterial translocation, which often occurs at the mesenteric lymph nodes, liver, and spleen, may activate an inflammatory response. The inflammatory mediators such as tumor necrosis factor-*α* (TNF-*α*), interleukin-1*β* (IL-1*β*), and IL-6 were released initially [6, 7]. TNF-*α* amplifies inflammatory cascades by activating macrophages/monocytes to secrete other inflammatory cytokines. The overproduction of inflammatory mediators induces endothelial and epithelial injury, vascular leakage, edema, and vasodilatation. In addition, the oxidative stress and mitochondrial dysfunction also exert vital roles in the organ damages and MODS [8–11]. The development of new therapeutic methods of anti-inflammatory, antioxidant, or regulating intestinal bacteria may reduce the incidence and mortality of sepsis.

Serotonin, or 5-hydroxytryptamine (5-HT), is an important regulatory factor in the gastrointestinal (GI) tract and other organ systems in addition to its role as a brain neurotransmitter [12]. The majority of peripheral 5-HT in the body is produced by the rate-limiting enzyme tryptophan hydroxylase 1 (TPH1). TPH1 knockout (TPH1−/−) mice show very low levels of circulating 5-HT but maintain normal levels of central 5-HT due to the blood-brain barrier [13–15]. Peripheral 5-HT is mainly stored in the platelets and released when activated. To date, 15 different 5-HT receptors (HTR) have been identified [16]. Binding to its receptors, peripheral 5-HT regulates diverse functions such as cardiac functions, platelet aggregation, bone development, and immune responses. The dysregulation of peripheral 5-HT is involved in the pathogenesis of irritable bowel syndrome (IBS), immune tolerance and inflammation, ischemia reperfusion injury, inflammatory bowel diseases, zymosan-induced MODS, acetaminophen-induced liver toxicity, acute pancreatitis, tumorigenesis of hepatocellular carcinoma (HCC), and so on [17–21].

It has been verified that gut microbiota regulates the host 5-HT biosynthesis and bacterial translocation contributes to the sepsis [22–24]. Besides, 5-HT facilitates the oxidative stress, nitrative stress, and lipid peroxidation in the pathological process of many diseases [25]. Furthermore, 5-HT has significant proinflammatory function in inflammation-linked diseases by regulating the release of inflammatory mediators and recruitment of inflammatory cells [16, 26]. Considering these facts, we speculated that 5-HT might participate in the process of sepsis. The previous studies showed that platelet-derived 5-HT promoted the recruitment of neutrophils in sites of acute inflammation in mice. Moreover, a sepsis-induced elevation in plasma 5-HT facilitated endothelial hyperpermeability [26–28]. However, there was no direct evidence whether 5-HT regulated anti-inflammatory, antioxidant, and bacterial production and drove mortality in sepsis. The main purpose of this study was to investigate the potential effects and mechanisms of 5-HT in the process of sepsis.

## 2. Materials and Methods

### 2.1. Experimental Animals

The study was conducted using male TPH1 knockout mice (TPH1−/− mice, kindly presented by Max Planck Institute for Molecular Genetics, Berlin, Germany) and wild-type C57BL/6 mice (4-5 weeks old, 20–26 g) (Animal Feeding Center of Xi'an Jiaotong University Health Science Center). The TPH1−/− mice were originated from C57BL/6 mice. The lack of peripheral 5-HT was the only difference between TPH1−/− mice and C57BL/6 mice. The characteristics of TPH1−/− mice had been described previously [15, 26, 29, 30]. Five mice were allocated to each cage. Moreover, the laboratory conditions were as follows: 23°C, 50% humidity, 12 h light daily, and the availability of sufficient food and water. The animal protocol was designed to minimize pain and discomfort to the animals. All animals were euthanized by isoflurane gas for tissue collection. All animal experiments were performed in accordance with the guidelines of the China Council on Animal Care and Use. All animal procedures performed in this study were reviewed, approved, and supervised by the Institutional Animal Care and Use Committee of the Ethics Committee of Xi'an Jiaotong University Health Science Center, China.

### 2.2. Experimental Design

The mice were randomly allocated into the following groups: (1) WT sham group: a sham laparotomy operation was performed on the WT mice. (2) KO sham group: a sham laparotomy operation was performed on TPH1 knockout mice. (3) WT-CLP sepsis group: the cecal ligation and puncture (CLP) with a 21-gauge needle was performed on WT mice. (4) KO-CLP sepsis group: the CLP with a 21-gauge needle was performed on TPH1 knockout mice. (5) WT+PCPA-CLP sepsis group: wild-type mice were pretreated with p-chlorophenylalanine (PCPA, TPH1 inhibitor, at a dose of 150 mg/kg/day for 3 days by subcutaneous injection before the CLP with a 21-gauge needle [20]). (6) KO+HTP-CLP sepsis group: TPH1 knockout mice were pretreated with 5-hydroxytryptophan (5-HTP) (precursor of 5-HT which can reload the 5-HT content, at a dose of 75 mg/kg/day for 3 days by subcutaneous injection before the CLP with a 21-gauge needle [20]). (7) WT-CLP septic shock group: the CLP with an 18-gauge needle was performed on WT mice. (8) KO-CLP septic shock group: the CLP with an 18-gauge needle was performed on TPH1 knockout mice. (9) WT+PCPA-CLP septic shock group: WT mice were pretreated with PCPA before the CLP with an 18-gauge needle. (10) KO+HTP-CLP septic shock group: TPH1 knockout mice were pretreated with 5-HTP before the CLP with an 18-gauge needle. In the experiment, the animals (*n* = 6 for the sham groups and *n* = 15 for the experimental groups) were randomly divided as described above and were monitored to assess mortality for 5 days in the CLP sepsis model and 3 days in the CLP septic shock model. The temperature and clinical score of each animal was measured as previously described [31].

### 2.3. Sepsis and Septic Shock Model: CLP

The mice were anesthetized with chloral hydrate (10 ml/kg, 4%). The abdomen was disinfected with iodine volts, and a 2 cm midline abdominal incision was conducted. The cecum was exposed, ligated below the ileocecal valve with a 3-0 silk, and then punctured with a 21-gauge needle for the sepsis model or an 18-gauge needle for the septic shock model. After the operation, the cecum was placed back to the original location. Saline (30 ml/kg) was used for anesthesia recovery in the incubator. The mice were returned to their cages until full recovery [32, 33]. Only abdominal incision was performed on the WT and KO sham group mice, exposed for 5 minutes and then closed in layers.

### 2.4. Histologic Analysis

Six hours and 24 h after the CLP and sham operations, respectively, tissue samples from the peritoneum, lung, liver, kidney, intestines, and hearts were collected for histologic analysis. The tissues were fixed in 10% formalin for 48 hours and then treated with paraffin embedding. Serial sections of 5 *μ*m thickness were obtained and stained with hematoxylin and eosin (H&E). Then, two researchers assessed the histologic changes in a double-blinded way to select a representative field for future use through the light microscope.

### 2.5. Quantification of Organ Function and Injury

At 6 h and 24 h after CLP, the mice were sacrificed to evaluate the acute peritonitis. Through a midline abdominal incision, 5 ml phosphate-buffered saline (PBS) was injected into the abdominal cavity. Then, all abdominal cavity liquid was collected to EP tubes. The exudate volume was calculated by subtracting the volume injected (5 ml) from the total volume. The mice serum was collected at 6 h and 24 h after the CLP. The liver and kidney injury was evaluated by measuring the level of serum alanine aminotransferase (ALT) and blood urea nitrogen (BUN). The activities of ALT and BUN were determined by an automated procedure in the Department of Inspection (The First Affiliated Hospital of Xi'an Jiaotong University). Lung dysfunction was assessed by measuring the W/D weight ratio. The superior and middle lobes of the right lung were gathered for the assessment of the lung W/D ratio. The lung samples were weighed immediately after collection, then placed into an incubator at 80°C for 48 h to dry and weighed again. The lung W/D ratio was calculated by the wet weight divided by the dry weight. Intestine dysfunction was assessed by measuring the villus height in H&E staining. The heart dysfunction was assessed by the ejection fraction (echocardiography).

### 2.6. Enzyme-Linked Immunosorbent Assays (ELISA)

The levels of serum TNF-*α*, IL-6, and IL-1*β* were measured with commercial ELISA kits according to the instructions of the manufacturer (Dakewe, Shenzhen, China).

### 2.7. Measurement of Peritoneal, Blood, and Lung Bacterial Loads

At 24 h after the CLP, the mice were sacrificed. The peritoneal fluid, blood, and lung tissues were collected. After diluted with phosphate-buffered saline (PBS), the peritoneal fluid, blood, and mixed liquid of the lung tissues were cultured overnight on blood-agar base plates (Becton Dickinson, USA) at 37°C. The colony-forming units (CFUs) of peritoneal, blood, and lung bacterial loads were calculated.

### 2.8. Immunofluorescence

At 24 h after the CLP, the fresh lung and liver tissues of mice were harvested for tissue slides. The lung and liver tissue slides were fixed in 10% formaldehyde and blocked with 10% normal donkey serum diluted in PBS containing 0.1% triton. Then, the slides were incubated with a polyclonal rabbit anti-MPO antibody (Santa Cruz Biotechnology, Inc., CA) diluted 1 : 200 in PBS for 1 hour at room temperature. The slides were washed and incubated with a donkey anti-rabbit antibody at 1 : 500 in PBS for 90 min at room temperature and counterstained with 4′6-diamidino-2-phenylindole (DAPI). The tissue sections were randomly screened (five fields/slide). Images were acquired using an inverted Leica CTR 6000 fluorescence microscope and merged with Leica Application Suite Advanced Fluorescence software (Leica UK, Milton Keynes).

### 2.9. Measurement of MPO Activity

The activity of MPO was measured using an activity assay kit (NanJing JianCheng Bioengineering). The liver and lung tissues were homogenized with PBS and centrifuged at 1500*g* for 15 min. The supernatants were obtained and stored at −80°C for the following analysis. The detection was conducted following the reference manual.

### 2.10. DHE Fluorescence Measurement

The fresh liver and lung tissues were collected and stored at −80°C. Five-millimeter thickness tissue sections were performed and incubated in Locke's buffer containing DHE concentration of 10 nM for 30 min. The lung tissue fluorescence (adopt excitation at 490 nm, emission at 610 nm) was observed by using fluorescence microscope.

### 2.11. Statistical Analysis

The measurement data were expressed as mean ± SD. Student's *t*-test was used for the comparison between the two groups. Kaplan-Meier curve was used for survival analysis and log-rank test for difference between the two groups. All tests were two-sided, and significance was accepted at *p* < 0.05. The statistical analyses were performed using GraphPad Prism software (version 6.0, GraphPad Software, Inc., La Jolla, CA, USA).

## 3. Results

### 3.1. Effect of PCPA and 5-HTP Treatment on the 5-HT Levels of WT and TPH1−/− Mice

To validate the role of 5-HT in CLP-induced sepsis, the PCPA (a classical TPH1 enzyme inhibitor) and 5-HTP (the precursor of 5-HT) were injected to mice 3 days before CLP operation. The plasma 5-HT levels were measured to confirm the effects of PCPA and 5-HTP in the WT and TPH1−/− mice. The results showed that plasma 5-HT levels in the WT mice treated with PCPA for 3 days significantly decreased compared with that in the WT mice treated with saline (306.0 ± 12.86 versus 3203.00 ± 170.563 ng/ml, *p* < 0.0001). Meanwhile, TPH1−/− mice treated with 5-HTP for 3 days could markedly replenish blood 5-HT in contrast to the TPH1−/− mice treated with saline (2544.60 ± 190.992 versus 287.850 ± 13.4185 ng/ml, *p* < 0.0001).

### 3.2. 5-HT Drives Mortality in Sepsis and Septic Shock

**Figure 1:**
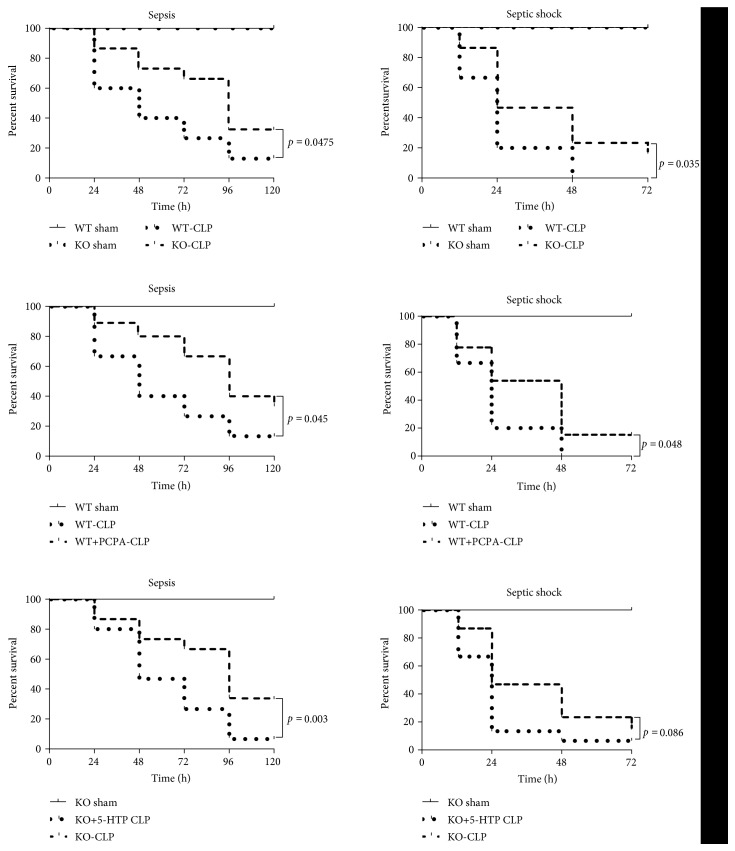
5-HT drives the survival rate in a sepsis and septic shock model in mice. Kaplan-Meier survival curve for mice 5 days after the CLP sepsis model and 3 days after the CLP septic shock model. PCPA, a TPH1-inhibitor, at a dose of 150 mg/kg/day for 3 days by subcutaneous injection before the CLP. 5-Hydroxytryptophan (5-HTP), a precursor of 5-HT that can reload the 5-HT content, at a dose of 75 mg/kg/day for 3 days by subcutaneous injection before CLP). (WT sham group and KO sham group, *n* = 6; WT-CLP groups, KO-CLP groups, WT+PCPA-CLP groups or KO+HTP-CLP groups, *n* = 15, *p* < 0.05 was statistically significant.)

The animals (*n* = 6 for sham groups and *n* = 15 for experimental groups) were randomly grouped and monitored to assess the 5-day survival rate in CLP-induced sepsis and the 3-day survival rate in CLP-induced septic shock (Figure 1). A log-rank test analysis of the 5-day survival curves in CLP sepsis showed that the survival rate of the KO-CLP group was significantly higher than that in the WT-CLP group (*p* = 0.0475). Furthermore, we conducted the experiment by adding PCPA and 5-HTP. The WT+PCPA-CLP sepsis group also showed an increased survival rate compared with the WT-CLP sepsis group (*p* = 0.045), while the survival rate of the KO+HTP-CLP sepsis group was lower than that of the KO-CLP sepsis group (*p* = 0.003). The mortality was revealed to be more serious in the CLP-induced septic shock model. Similar results were obtained for the septic shock model, demonstrating that the survival rate of the KO-CLP group was significantly higher than that of the WT-CLP group (*p* = 0.035); and PCPA had a protective effect on the WT+PCPA-CLP septic shock group, which had a higher survival rate than the WT-CLP septic shock group 3 days after the CLP (*p* = 0.048). However, there was no difference between the KO+5-HTP CLP septic shock group and the KO-CLP group (*p* = 0.086). The above results demonstrated that 5-HT deficiency provided a significant level of protection.

### 3.3. Effect of 5-HT on Sepsis Manifestation

**Figure 2:**
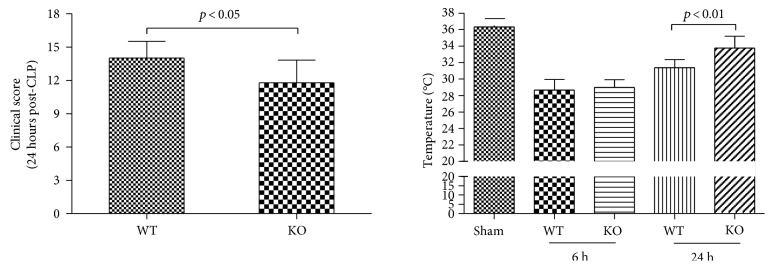
Lack of 5-HT reduces the clinical score and retains low body temperature in CLP-induced sepsis in mice. The clinical score was assessed at 24 h, and the temperatures of the mice were measured at 6 h and 24 h after CLP-induced sepsis (*n* = 15, mean ± SD, clinical score, *p* < 0.05 versus the WT-CLP group; temperature, *p* < 0.01 versus the WT-CLP group 24 h after CLP-induced sepsis).

Two researchers, in a double-blinded study, evaluated the clinical score of the sepsis severity 24 hours after the CLP. The KO-CLP sepsis group received a lower score than the WT-CLP sepsis group (*p* < 0.05). Meanwhile, the body temperature of the mice in the KO-CLP sepsis group was higher than the WT-CLP sepsis group and was much closer to the normal body temperature after 24 hours (*p* < 0.01) (Figure 2).

### 3.4. Effect of 5-HT on Tissue Histopathology Change and Organ Dysfunction in Sepsis

**Figure 3:**
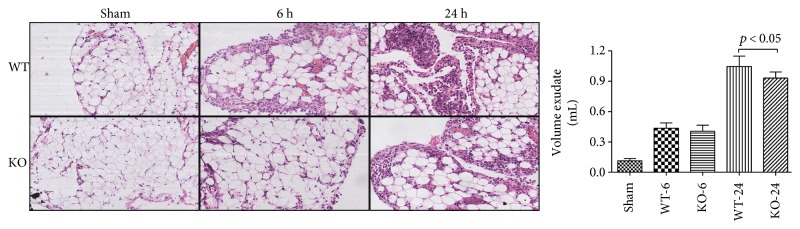
Lack of 5-HT alleviates peritonitis in CLP-induced sepsis in mice. The peritoneum tissue were harvested at 6 h or 24 h after CLP administration. The visceral peritoneum tissue sections were stained with H&E. Original magnification 200x. Figures are representative of at least three experiments performed on different experimental days. The amount of exudate was calculated for assessment of acute peritonitis (*n* = 6, amount of exudate: mean ± SD, *p* < 0.05 versus the WT-CLP group at 6 h after the CLP administration).

**Figure 4:**
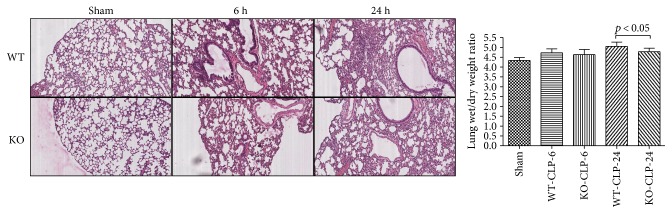
Lack of 5-HT alleviates histopathology damage and organ dysfunction in the lung in CLP-induced sepsis in mice. The lung tissues were harvested at 6 h or 24 h after the CLP administration. The lung tissue sections were stained with H&E. Original magnification 200x. In the lung, the inflammatory infiltration of neutrophils, macrophages, and plasma cells in the WT-CLP sepsis group was more serious than in the KO-CLP sepsis group. The figures are representative of at least three experiments performed on different experimental days. The lung wet/dry weight ratio was calculated as an assessment of lung dysfunction (*n* = 6, lung wet/dry weight ratios: calculated by dividing the wet weight by the dry weight. Mean ± SD, *p* < 0.05 versus the WT-CLP group at 6 after the CLP administration).

**Figure 5:**
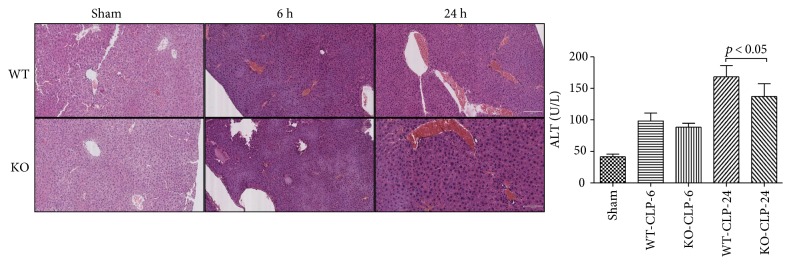
Lack of 5-HT alleviates histopathology damage and organ dysfunction in the liver in CLP-induced sepsis in mice. The animal blood samples and liver tissues were harvested at 6 h or 24 h after the CLP administration. H&E staining was conducted as described above. In the liver, the WT mice showed a marked congestion, inflammatory cell infiltration, necrosis, and degeneration. The KO-CLP mice had alleviated histopathological changes. ALT was calculated as an assessment of liver dysfunction (*n* = 6, ALT: mean ± SD, *p* < 0.05 versus the WT-CLP group at 24 h after the CLP administration).

**Figure 6:**
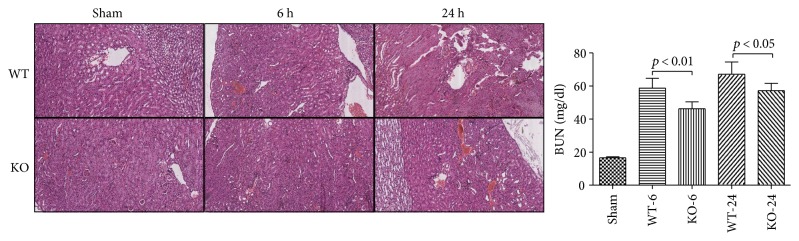
Lack of 5-HT alleviates histopathology damage and organ dysfunction in the kidney in CLP-induced sepsis in mice. The animal blood samples and the kidney tissues were harvested at 6 h or 24 h after the CLP administration. H&E staining was conducted as described above. In the kidney, the mice in the KO-CLP sepsis group had less inflammatory cell infiltration, necrosis, and degeneration. The BUN was calculated as an assessment of renal dysfunction (*n* = 6, BUN: mean ± SD, *p* < 0.01 versus the WT-CLP group at 6 h after the CLP administration; *p* < 0.05 versus the WT-CLP group at 24 h after the CLP administration).

**Figure 7:**
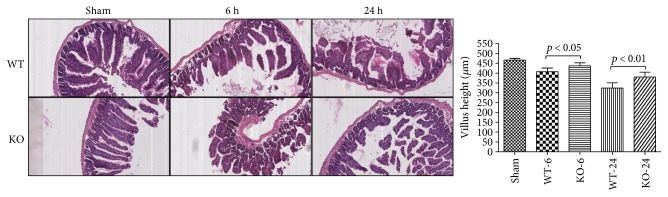
Lack of 5-HT alleviates the histopathology damage and organ dysfunction in the intestine in CLP-induced sepsis in mice. The intestine tissues were harvested at 6 h or 24 h after CLP administration. H&E staining was conducted as described above. In the intestine sections, the KO-CLP mice showed a reduced infiltration of inflammatory cells, edema in the space bounded by the villus, and separation of the epithelium from the basement membrane. The villus height was measured as an assessment of intestine dysfunction (*n* = 6, villus height: mean ± SD, *p* < 0.05 versus the WT-CLP group at 6 h after the CLP administration; *p* < 0.01 versus the WT-CLP group at 24 h after the CLP administration).

**Figure 8:**
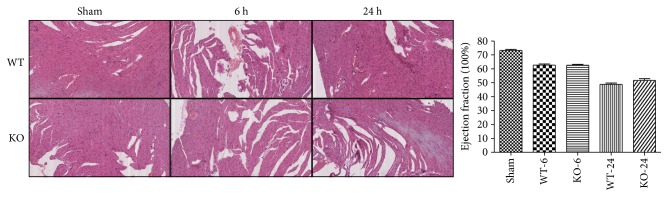
Lack of 5-HT alleviates the histopathology damage and organ dysfunction in the heart in CLP-induced sepsis in mice. The animal heart tissues were harvested at 6 h or 24 h after CLP administration. H&E staining was conducted as described above. In the heart, the KO-CLP mice showed less inflammatory cell infiltration, necrosis, and degeneration than the WT-CLP mice. The ejection fraction was measured as an assessment of heart dysfunction (*n* = 6, ejection fraction: mean ± SD; there has no significant difference at both 6 h and 24 h after the CLP administration).

At 6 h and 24 h after the CLP administration, a histological evaluation of the lung, liver, kidney, intestine sections, and heart exhibited several marked pathological changes and organ dysfunctions. In the lung, the histologic analysis revealed that an inflammatory infiltration of neutrophils, macrophages, and plasma cells in the WT-CLP sepsis group was more serious than that in the KO-CLP sepsis group, especially at 6 h after the CLP. The WT-CLP mice also showed more severe peritonitis compared with the KO-CLP mice at 24 h (*p* < 0.05) (Figure 3). Moreover, the WT-CLP mice showed a higher lung W/D weight ratio compared with the KO-CLP mice at 6 h (*p* < 0.05) (Figure 4). In the liver, CLP induced marked congestion, inflammatory cell infiltration, necrosis, and degeneration. The KO-CLP mice alleviated histopathological changes and had a lower level of ALT at 24 h (*p* < 0.05) (Figure 5). In the kidney, the mice in the KO-CLP sepsis group had better results for inflammatory cell infiltration and necrosis as well as lower levels of BUN in contrast to the WT-CLP sepsis group (*p* < 0.05) (Figure 6). In the intestine sections, the massive inflammatory cell infiltration, edema, and separation of the epithelium from the basement membrane were observed after the CLP treatment. In addition, the villus height suffered less damages in the KO-CLP mice (*p* < 0.05) (Figure 7). In the heart, the KO-CLP mice showed less inflammatory cell infiltration, necrosis, and degeneration than the WT-CLP mice; whereas, the ejection fractions were not significantly different (Figure 8). Based on the evidences above, we concluded that 5-HT played detrimental roles in the organ functions.

### 3.5. Effect of 5-HT on Serum Cytokines and Bacterial Loads

**Figure 9:**
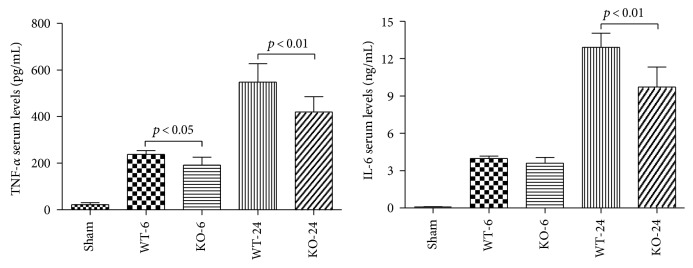
Lack of 5-HT decreases the level of serum cytokines in CLP-induced sepsis. The levels of serum TNF-*α* and IL-6 were measured with commercial ELISA kits according to the instructions of the manufacturer. A lack of 5-HT reduced the serum TNF-*α* and IL-6 concentrations (*n* = 6, mean ± SD, TNF-*α*: *p* < 0.05 versus the WT-CLP group at 6 h after the CLP administration. *p* < 0.01 versus the WT-CLP group at 24 h after the CLP administration; IL-6: *p* < 0.01 versus the WT-CLP group at 24 h after the CLP administration).

The inflammatory response plays a fatal role in the process of sepsis. Serum cytokines were assessed by determining the serum levels of the TNF-*α* and IL-6 (Figure 9). A marked increase of TNF-*α* and IL-6 secretion was observed in the CLP sepsis group compared with the sham group (*p* < 0.05). Moreover, a significant inhibition of TNF-*α* was discovered in the KO-CLP sepsis group in contrast to the WT-CLP sepsis group both at 6 h and 24 h after the CLP (*p* < 0.05), while the KO-CLP mice had a lower level of IL-6 at 24 h rather than at 6 h after the CLP compared with the WT-CLP mice (*p* < 0.01). In conclusion, 5-HT increased serum cytokine secretion, such as TNF-*α* and IL-6, and aggravated the mortality of sepsis by an excessive inflammatory response.

**Figure 10:**
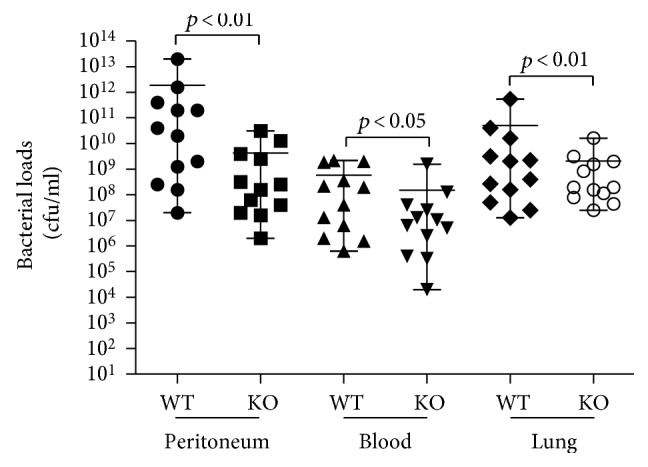
Lack of 5-HT decreases the bacterial loads in CLP-induced sepsis. The mice were anesthetized, and the peritoneal fluid, blood, and lung tissue were collected to measure the bacterial loads 24 h after the CLP. The bacterial loads were significantly decreased in the KO-CLP mice compared with the WT-CLP mice in the peritoneal fluid, blood, and lung tissue. All data are expressed as the mean ± SD (*n* = 6, *p* < 0.05 versus the WT-CLP group).

Bacteria and their metabolic products are the initial cause of sepsis. The peritoneal fluid, blood, and lung tissues were collected for measuring the bacterial loads (Figure 10). We discovered that the bacterial loads were significantly decreased in the KO-CLP mice compared with the WT-CLP mice in the peritoneal fluid, blood, and lung tissues (*p* < 0.05).

### 3.6. Effect of 5-HT on Oxidative Stress in Sepsis

**Figure 11:**
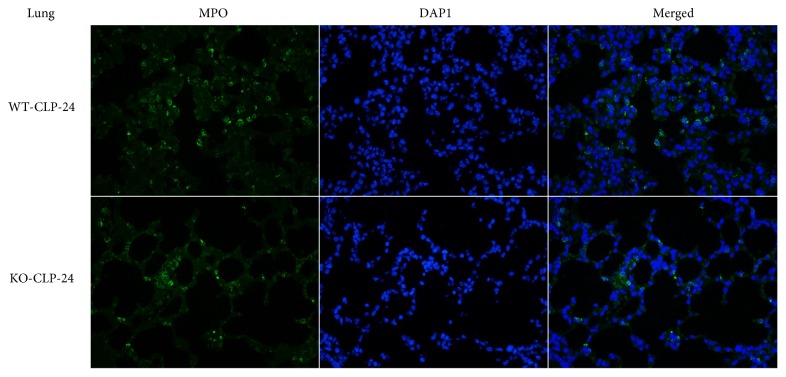
Lack of 5-HT decreases the level of MPO in the lung in CLP-induced sepsis. MPO staining of the lung tissue at 24 h after CLP administration. The MPO-stained cells (green), the corresponding nuclear counterstaining (blue), and both channels merged display the relative change in the level of MPO observed in a typical field. The MPO staining was significantly decreased in the KO-CLP sepsis group compared to the WT-CLP sepsis group in the lung at 24 h after CLP. Original magnifications, ×400.

**Figure 12:**
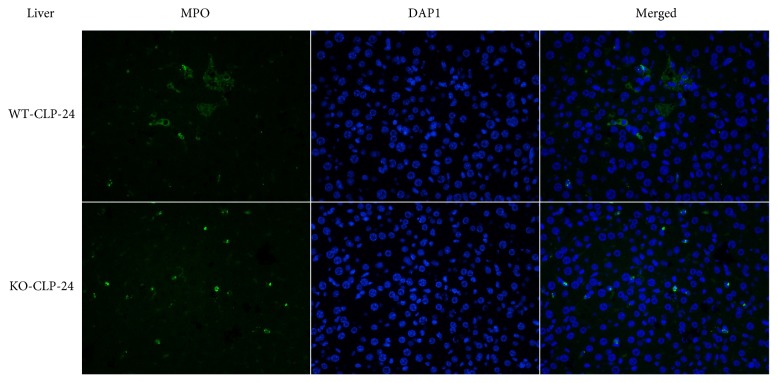
Lack of 5-HT decreases the level of MPO in the liver in CLP-induced sepsis. MPO staining of the liver tissue at 24 h after CLP administration. The MPO-stained cells (green), the corresponding nuclear counterstaining (blue), and both channels merged display the relative change in the level of MPO observed in a typical field. The MPO staining was significantly decreased in the KO-CLP sepsis group compared to the WT-CLP sepsis group in the liver at 24 h after the CLP. Original magnifications, ×400.

**Figure 13:**
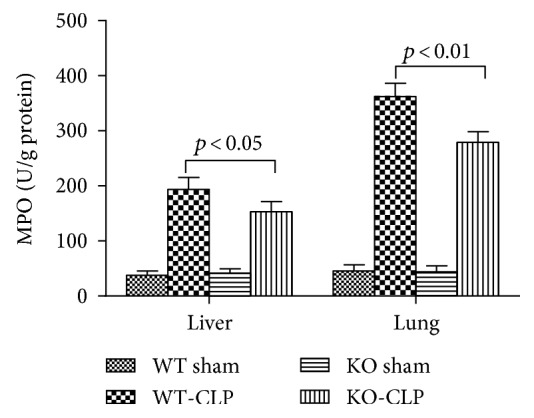
Lack of 5-HT decreases the level of MPO in CLP-induced sepsis. The activities of myeloperoxidase (MPO) in the liver and lung tissues were measured using activity assay kits. All data are expressed as the mean ± SD. *p* < 0.05 versus the WT-CLP group.

**Figure 14:**
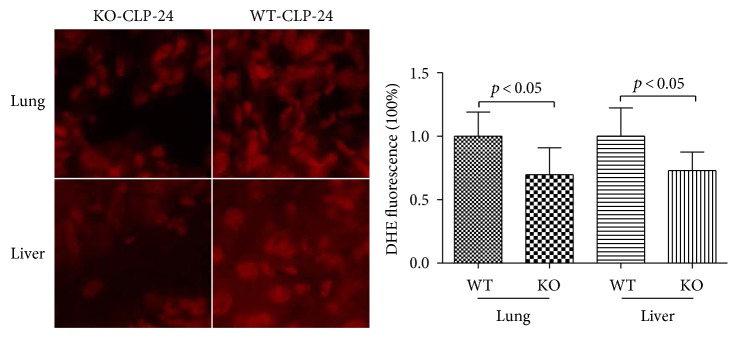
Lack of 5-HT decreases the level of ROS in CLP-induced sepsis. DHE fluorescence intensity was used to detect ROS after CLP in the lung and liver. The KO-CLP sepsis group had a lower level of fluorescence intensity than the WT-CLP sepsis group in both the liver and lung. All data are expressed as the mean ± SD. *p* < 0.05 versus the WT-CLP group.

MPO is a marker of neutrophil infiltration as well as a marker of oxidative stress. The level of MPO, determined by the MPO staining, was significantly lower in the KO-CLP sepsis group than in the WT-CLP sepsis group in the liver and lung at 24 h after the CLP (Figures 11 and 12). The MPO quantification results exhibited that the level of MPO markedly increased after the CLP treatment in contrast to the sham mice. In addition, the level of MPO in the KO-CLP sepsis group was significantly lower than in the WT-CLP sepsis group in the lung and liver (*p* < 0.05) (Figure 13). DHE fluorescence was conducted to measure ROS, and there was a lower level of fluorescence intensity in the KO-CLP sepsis group than in the WT-CLP sepsis group in both the liver and lung, which revealed that the KO-CLP mice had a reduced level of ROS compared with the WT-CLP mice (Figure 14).

## 4. Discussion

In this study, a model of sepsis and septic shock was established in mice by CLP and the influences of 5-HT on survival rate, sepsis manifestation, organ dysfunction, serum cytokines, bacterial loads, and oxidative stress were assessed to investigate whether 5-HT drives mortality and the potential mechanism in the process of sepsis. With this work, we demonstrated that 5-HT markedly increased mortality in sepsis and septic shock. The study further exhibited that 5-HT exacerbated the general manifestation as well as caused histological damages in the lung, liver, kidney, intestinal tissue, and heart. The mechanisms that 5-HT aggravated the sepsis might include the following: (1) the increasing secretion of serum cytokines, such as TNF-*α* and IL-6, and an excessive inflammatory response; (2) the acceleration of bacterial translocation; and (3) the promotion of oxidative stress, for instance, MPO and ROS. All the evidence illustrated that 5-HT could increase the risk of sepsis. In contrast, the5-HT deficiency provided a significant level of protection. Bacteria and their metabolic products caused an uncontrollable inflammatory response and played a crucial role in sepsis. The relationship between bacterial translocation and sepsis seemed to be a positive regulation. The previous study proves that there is a close association between bacterial translocation and gut-derived sepsis and bacterial translocation has been put forward as a concept to explain sepsis without an infectious focus [7, 34]. Meanwhile, sepsis accelerates bacterial translocation by destroying the intestinal barrier and increasing intestinal permeability [35–37]. It has been demonstrated that 5-HT exacerbated DSS-induced colitis by upregulating MMP-3 and MMP-9 expression in mice [38]. In our study, we discovered that the absence of 5-HT reduced bacterial loads in the peritoneal fluid, blood, and lung tissue in sepsis. 5-HT might increase intestinal permeability and play a role in promoting bacterial translocation.

Our findings showed that 5-HT exacerbated the histological damages in the lung, liver, kidney, intestinal tissue, and heart, which resulted in organ dysfunction. A recent research claimed that a sepsis-induced elevation in plasma 5-HT facilitated the endothelial hyperpermeability [28]. In this situation, the organs were susceptible to injuries from bacteria, serum cytokines, and oxidative stress. 5-HT also promoted the recruitment of neutrophils and regulated immune tolerance and inflammation [26, 39, 40]. Our study revealed that 5-HT aggravated the histological changes, including congestion, inflammatory cell infiltration, necrosis, and degeneration.

TNF-*α* is released by macrophages in response to infection, stimulates the production of downstream cytokines, such IL-6 and IL-8, and plays a significant role in activating the cytokine cascade [41, 42]. In the previous study, the levels of TNF-*α* and IL-6 in the plasma were also substantially increased in CLP-induced sepsis [43]. Our study indicated that the levels of TNF-*α* were decreased in the 5-HT deficiency group at both 6 h and 24 h. However, the absence of 5-HT inhibited the level of IL-6 at 24 h but not at 6 h after the CLP. The possible reason why the KO-CLP mice and the WT-CLP mice showed no differences 6 h after the CLP administration might be that IL-6 was not massively released at the early stage of inflammation.

The effect of oxidative stress has been widely studied in previous studies [10, 44, 45]. During sepsis, ROS and MPO production exceeds antioxidant defenses and leads to a state of oxidative stress that fuels inflammation and causes direct mitochondrial damages, which is suggested to play a central role in sepsis-induced organ dysfunction [46, 47]. MPO is a marker of oxidative stress except for neutrophil infiltration. In addition, therapeutic strategies to protect the mitochondria by decreasing oxidative stress during sepsis have been recognized [48–50]. Our study showed that the levels of ROS detected by DHE fluorescence and MPO by immunofluorescence were markedly increased after the CLP treatment and decreased in the KO-CLP mice compared with the WT-CLP mice in the lung and liver. We concluded that 5-HT aggravated sepsis by facilitating the occurrence of oxidative stress that fueled inflammation.

In this study, we discovered that 5-HT drove mortality and exacerbated organ dysfunction through the promotion of serum cytokines and bacterial loads and by facilitating oxidative stress in the process of sepsis. This study proved that 5-HT regulated sepsis progression and might be a new therapeutic target for sepsis. However, the mechanism of increasing bacterial loads induced by bacterial translocation has no direct evidence and further studies are required. Whether the mechanism of endothelial hyperpermeability and aggravating injury plays a key role in histological damages and organ dysfunctions induced by 5-HT needs further studies. Moreover, prospective clinical studies are needed to evaluate whether the level of 5-HT relates to the process of sepsis.

## 5. Conclusions

In conclusion, the results of the present study demonstrated that 5-HT markedly drove mortality and exacerbated organ dysfunctions in sepsis. The potential mechanisms might be that 5-HT facilitated the inflammation, bacterial loads, and oxidative stress. These findings indicated that 5-HT played a key role in the process of sepsis and might be a potential therapeutic option in sepsis.

## References

[1] Singer M., Deutschman C. S., Seymour C. W. (2016). The third international consensus definitions for sepsis and septic shock (sepsis-3). *Jama*.

[2] Seymour C. W., Liu V. X., Iwashyna T. J. (2016). Assessment of clinical criteria for sepsis: for the third International consensus definitions for sepsis and septic shock (sepsis-3). *Jama*.

[3] Rhodes A., Evans L. E., Alhazzani W. (2017). Surviving sepsis campaign: international guidelines for management of sepsis and septic shock: 2016. *Intensive Care Medicine*.

[4] Rossaint J., Zarbock A. (2015). Pathogenesis of multiple organ failure in sepsis. *Critical Reviews in Immunology*.

[5] Sganga G. (2015). Surgical sepsis. *Urologia*.

[6] Lamprecht G., Heininger A. (2012). Current aspects of sepsis caused by bacterial translocation. *Zentralblatt für Chirurgie*.

[7] Deitch E. A. (2012). Gut-origin sepsis: evolution of a concept. *The Surgeon*.

[8] Cojocaru I. M., Musuroi C., Iacob S., Cojocaru M. (2003). Investigation of TNF-alpha, IL-6, IL-8 and of procalcitonin in patients with neurologic complications in sepsis. *Romanian Journal of Internal Medicine*.

[9] Erbas O., Taskiran D. (2014). Sepsis-induced changes in behavioral stereotypy in rats; involvement of tumor necrosis factor-alpha, oxidative stress, and dopamine turnover. *The Journal of Surgical Research*.

[10] Quoilin C., Mouithys-Mickalad A., Lecart S., Fontaine-Aupart M. P., Hoebeke M. (2014). Evidence of oxidative stress and mitochondrial respiratory chain dysfunction in an in vitro model of sepsis-induced kidney injury. *Biochimica et Biophysica Acta*.

[11] Aksoy A. N., Toker A., Celik M., Aksoy M., Halıcı Z., Aksoy H. (2014). The effect of progesterone on systemic inflammation and oxidative stress in the rat model of sepsis. *Indian Journal of Pharmacology*.

[12] Mohammad-Zadeh L. F., Moses L., Gwaltney-Brant S. M. (2008). Serotonin: a review. *Journal of Veterinary Pharmacology and Therapeutics*.

[13] Cote F., Thevenot E., Fligny C. (2003). Disruption of the nonneuronal tph1 gene demonstrates the importance of peripheral serotonin in cardiac function. *Proceedings of the National Academy of Sciences of the United States of America*.

[14] Choi K. Y., Yoon H. K., Kim Y. K. (2010). Association between serotonin-related polymorphisms in 5HT2A, TPH1, TPH2 genes and bipolar disorder in Korean population. *Psychiatry Investigation*.

[15] Walther D. J., Peter J. U., Bashammakh S. (2003). Synthesis of serotonin by a second tryptophan hydroxylase isoform. *Science*.

[16] Shajib M. S., Khan W. I. (2015). The role of serotonin and its receptors in activation of immune responses and inflammation. *Acta Physiologica (Oxford, England)*.

[17] Olivier B. (2015). Serotonin: a never-ending story. *European Journal of Pharmacology*.

[18] Namkung J., Kim H., Park S. (2015). Peripheral serotonin: a new player in systemic energy homeostasis. *Molecules and Cells*.

[19] de Las Casas-Engel M., Corbi A. L. (2014). Serotonin modulation of macrophage polarization: inflammation and beyond. *Advances in Experimental Medicine and Biology*.

[20] Zhang J., Pang Q., Song S. (2015). Role of serotonin in MODS: deficiency of serotonin protects against zymosan-induced multiple organ failure in mice. *Shock*.

[21] Zhang J., Song S., Pang Q. (2015). Serotonin deficiency exacerbates acetaminophen-induced liver toxicity in mice. *Scientific Reports*.

[22] Yano J. M., Yu K., Donaldson G. P. (2015). Indigenous bacteria from the gut microbiota regulate host serotonin biosynthesis. *Cell*.

[23] Mawe G. M., Hoffman J. M. (2013). Serotonin signalling in the gut—functions, dysfunctions and therapeutic targets. *Nature Reviews. Gastroenterology & Hepatology*.

[24] Hunninghake G. W., Doerschug K. C., Nymon A. B., Schmidt G. A., Meyerholz D. K., Ashare A. (2010). Insulin-like growth factor-1 levels contribute to the development of bacterial translocation in sepsis. *American Journal of Respiratory and Critical Care Medicine*.

[25] Nocito A., Dahm F., Jochum W. (2007). Serotonin mediates oxidative stress and mitochondrial toxicity in a murine model of nonalcoholic steatohepatitis. *Gastroenterology*.

[26] Duerschmied D., Suidan G. L., Demers M. (2013). Platelet serotonin promotes the recruitment of neutrophils to sites of acute inflammation in mice. *Blood*.

[27] Farand P., Hamel M., Lauzier F., Plante G. E., Lesur O. (2006). Review article: organ perfusion/permeability-related effects of norepinephrine and vasopressin in sepsis. *Canadian Journal of Anaesthesia*.

[28] Li Y., Hadden C., Cooper A. (2016). Sepsis-induced elevation in plasma serotonin facilitates endothelial hyperpermeability. *Scientific Reports*.

[29] Lang P. A., Contaldo C., Georgiev P. (2008). Aggravation of viral hepatitis by platelet-derived serotonin. *Nature Medicine*.

[30] Lesurtel M., Graf R., Aleil B. (2006). Platelet-derived serotonin mediates liver regeneration. *Science*.

[31] Weber G. F., Chousterman B. G., He S. (2015). Interleukin-3 amplifies acute inflammation and is a potential therapeutic target in sepsis. *Science*.

[32] Hu B., Li Y., Gao L. (2017). Hepatic induction of fatty acid binding protein 4 plays a pathogenic role in sepsis in mice. *American Journal of Pathology*.

[33] Lilley E., Armstrong R., Clark N. (2015). Refinement of animal models of sepsis and septic shock. *Shock*.

[34] Gennari R., Alexander J. W., Eaves-Pyles T. (1995). Effect of different combinations of dietary additives on bacterial translocation and survival in gut-derived sepsis. *JPEN Journal of Parenteral and Enteral Nutrition*.

[35] Vaishnavi C. (2013). Translocation of gut flora and its role in sepsis. *Indian Journal of Medical Microbiology*.

[36] Tsujimoto H., Ono S., Mochizuki H. (2009). Role of translocation of pathogen-associated molecular patterns in sepsis. *Digestive Surgery*.

[37] Denlinger L. C. (2001). Low-dose prostacyclin reverses endotoxin-induced intestinal vasoconstriction: potential for the prevention of bacterial translocation in early sepsis. *Critical Care Medicine*.

[38] Chen M., Gao L., Chen P. (2016). Serotonin-exacerbated DSS-induced colitis is associated with increase in MMP-3 and MMP-9 expression in the mouse colon. *Mediators of Inflammation*.

[39] Mauler M., Bode C., Duerschmied D. (2016). Platelet serotonin modulates immune functions. *Hämostaseologie*.

[40] Nowak E. C., de Vries V. C., Wasiuk A. (2012). Tryptophan hydroxylase-1 regulates immune tolerance and inflammation. *The Journal of Experimental Medicine*.

[41] Cavaillon J. M., Adib-Conquy M., Fitting C., Adrie C., Payen D. (2003). Cytokine cascade in sepsis. *Scandinavian Journal of Infectious Diseases*.

[42] Ertel W., Krombach F., Kremer J. P. (1993). Mechanisms of cytokine cascade activation in patients with sepsis: normal cytokine transcription despite reduced CD14 receptor expression. *Surgery*.

[43] de Jong H. K., van der Poll T., Wiersinga W. J. (2010). The systemic pro-inflammatory response in sepsis. *Journal of Innate Immunity*.

[44] Lowes D. A., Webster N. R., Murphy M. P., Galley H. F. (2013). Antioxidants that protect mitochondria reduce interleukin-6 and oxidative stress, improve mitochondrial function, and reduce biochemical markers of organ dysfunction in a rat model of acute sepsis. *British Journal of Anaesthesia*.

[45] Galley H. F. (2011). Oxidative stress and mitochondrial dysfunction in sepsis. *British Journal of Anaesthesia*.

[46] Liu C. H., Zhang W. D., Wang J. J., Feng S. D. (2016). Senegenin ameliorate acute lung injury through reduction of oxidative stress and inhibition of inflammation in cecal ligation and puncture-induced sepsis rats. *Inflammation*.

[47] Zolali E., Asgharian P., Hamishehkar H., Kouhsoltani M., Khodaii H., Hamishehkar H. (2015). Effects of gamma oryzanol on factors of oxidative stress and sepsis-induced lung injury in experimental animal model. *Iranian Journal of Basic Medical Sciences*.

[48] Cassol-Jr O. J., Comim C. M., Silva B. R. (2010). Treatment with cannabidiol reverses oxidative stress parameters, cognitive impairment and mortality in rats submitted to sepsis by cecal ligation and puncture. *Brain Research*.

[49] Chen Y. J., Gong C. L., Tan F., Zhou S. L. (2015). Pretreatment with dexmedetomidine ameliorates renal inflammation and oxidative stress in rats with lipopolysaccharide-induced sepsis and acute kidney injury. *Nan Fang Yi Ke da Xue Xue Bao*.

[50] Gerin F., Sener U., Erman H. (2016). The effects of quercetin on acute lung injury and biomarkers of inflammation and oxidative stress in the rat model of sepsis. *Inflammation*.

